# Associations of Obesity with Function and Patient-Reported Outcomes Among Rural Advanced Cancer Patients: A Cross-Sectional Analysis of the Nurse AMIE Randomized Controlled Trial

**DOI:** 10.3390/cancers18010006

**Published:** 2025-12-19

**Authors:** Samantha J. Werts-Pelter, Clair Smith, Stephen Baker, Charity G. Patterson, Nicole Stout, Jennifer Moss, William A. Calo, Shawna E. Doerksen, Kathryn H. Schmitz

**Affiliations:** 1 Division of Hematology Oncology, Department of Medicine, School of Medicine, University of Pittsburgh, UPMC Hillman Cancer Center, Pittsburgh, PA 15232, USA; wertspelters@upmc.edu (S.J.W.-P.); bakers27@upmc.edu (S.B.); doerksens@upmc.edu (S.E.D.); 2 Department of Physical Therapy, School of Health and Rehabilitation Sciences Data Center, University of Pittsburgh, Pittsburgh, PA 15219, USA; cns45@pitt.edu (C.S.); cgp22@pitt.edu (C.G.P.); 3 American Cancer Society, Atlanta, GA 30303, USA; nicole.stout@cancer.org; 4 Department of Family and Community Medicine, Penn State College of Medicine, The Pennsylvania State University, Hershey, PA 17036, USA; jmoss1@pennstatehealth.psu.edu; 5 Department of Public Health Sciences, Penn State College of Medicine, The Pennsylvania State University, Hershey, PA 17033, USA; wcalo@pennstatehealth.psu.edu

**Keywords:** rural health, physical function, health-related quality of life, symptom management, cancer care

## Abstract

**Background/Objectives:** Obesity is a common comorbidity but there remains limited understanding on how higher obesity rates in rural areas may impact physical function decline and other health domains among cancer patients. This study addresses this gap by examining the association between body mass index (BMI) and physical function among a cohort of rural advanced cancer patients. **Methods:** This cross-sectional analysis uses baseline data from the Nurse AMIE trial (NCT04673019). Individuals were categorized as ‘normal weight’ (BMI ≤ 25 kg/m^2^), ‘overweight’ (BMI > 25 to 30 kg/m^2^), and ‘obese’ (BMI > 30 kg/m^2^). Objective physical function was measured by the Short Physical Performance Battery (SPPB) and subjective physical function and health domains were measured using surveys (PROMIS; SF-36). **Results:** Of 348 patients included, 88 (25.3%) were classified as ‘normal weight’, 107 (30.7%) as ‘overweight’, and 153 (44.0%) as ‘obese’. Average age was 64.8 years (SD = 12.2), 46% (n = 160) were female, 95% were white (n = 331), and 52% (n = 182) were Stage 4. Total SPPB scores revealed poorer functioning with higher BMI (M ± SD: BMI ≤ 25 kg/m^2^: 9.1 ± 2.3; BMI > 25–30 kg/m^2^: 8.3 ± 3.1; BMI > 30 kg/m^2^: 8.1 ± 2.8; *p* = 0.04). Similarly, scores from the SF-36 revealed subjective physical function was lower with higher BMI (BMI ≤ 25 kg/m^2^: 57.9 ± 29.1; BMI > 25–30 kg/m^2^: 53.7 ± 28.0; BMI > 30 kg/m^2^: 47.6 ± 27.6; *p* = 0.004). Participants reported lower levels of energy and greater fatigue with higher BMI (BMI ≤ 25 kg/m^2^: 49.8 ± 26.1; BMI > 25–30 kg/m^2^: 45.1 ± 24.6; BMI > 30 kg/m^2^: 40.7 ± 22.6; *p* = 0.01). **Conclusions:** Higher BMI is associated with poorer physical function and increased fatigue among rural advanced cancer patients, highlighting the need for supportive care related to physical function in this at-risk group.

## Simple Summary

The relationship between obesity and physical function among rural cancer patients is not well understood. High prevalence of obesity in rural areas may have a detrimental effect on physical functioning and health-related quality of life. This analysis examined the association between obesity and subjective and objective physical function, identifying that higher obesity levels may be correlated with poorer physical function among rural cancer survivors. These findings suggest that additional supportive care may be needed to support physical function among rural advanced cancer patients experiencing obesity.

## 1. Introduction

Obesity, defined as a body mass index (BMI) greater than 30 kg/m^2^, is a growing epidemic in the United States and globally [1,2,3]. Strong evidence indicates that being overweight (BMI > 25 kg/m^2^) is associated with an increased risk of 13 types of cancer including breast, colorectal, pancreatic, and liver [4,5]. Obesity is not only associated with an increased risk of developing cancer, but may also be linked to recurrence, increased treatment-related adverse effects, and decreased overall survival [6,7,8,9]. Evidence shows that obesity is associated with a 14% increased risk of overall mortality, 17% increased risk of cancer-specific mortality, and 13% increased risk of recurrence [6]. Treatment-related adverse effects that have been shown to increase with obesity include, but are not limited to, lymphedema, chemotherapy-induced peripheral neuropathy, and cardiotoxicity [10,11,12]. The dual burden of obesity and cancer requires additional research to understand treatment-related and long-term side effects and the impact on health-related quality of life.

In addition to treatment-related adverse effects, obesity may cause increased inflammation and physical deconditioning that compound the effect of cancer-related decline in function and energy [5]. Higher BMI has been shown to correlate with decreased upper- and lower-body function [13,14]. It has been hypothesized that higher levels of obesity may contribute to poor function by limiting mobility and flexibility, increasing chronic pain, and development of cardiovascular disease and arthritis [15,16,17]. Unfortunately for cancer patients and survivors, this combination of inflammation and reduced physical reserve may magnify pain, delay recovery, and diminish overall health-related quality of life [18,19,20]. Previous research has demonstrated a correlation between higher BMI and poorer quality of life, more comorbidities, and poorer physical functioning in cancer patients [19,20,21]. Additional work is needed to understand effects in those diagnosed with advanced cancer who may be facing a more difficult treatment course or who may have more complex symptom profiles that also contribute to poorer functional abilities.

The prevalence of obesity is not evenly distributed throughout the country. It has been shown that the prevalence of obesity is higher in rural (36%) versus urban areas (30%) [22,23]. For those diagnosed with cancer in rural areas, the disparity in obesity prevalence is just one of several risk factors leading to poorer overall survival, functional limitations, and lower health-related quality of life [24,25]. Rural populations face limited access to high quality cancer prevention and treatment services, higher prevalence of advanced stage at diagnosis, and greater symptom burden [26,27,28]. To best support the health and long-term outcomes for rural cancer patients, there is a need for focused research to understand the intersection between obesity and symptom burden, quality of life, and physical function. We sought to explore these associations using baseline data from the Nurse AMIE trial conducted among rural advanced cancer patients.

## 2. Materials and Methods

Nurse AMIE is a randomized controlled trial examining the effect of a tablet-based supportive care intervention on symptom management and overall survival among advanced cancer patients living in rural areas. Information about the study protocol and aims has been previously published [29]. This analysis uses baseline data from the Nurse AMIE trial to describe the association between obesity and objective and subjective physical function and patient-reported health.

Eligibility for the Nurse AMIE trial included diagnosis with stage 3 or 4 cancer of any tumor site or a cancer deemed ‘advanced’ by a clinician for those that are not staged. Participants must have lived in a rural area of Pennsylvania or West Virginia defined as residing in a county with a Rural Urban Continuum Code (RUCC) of 4–9 [30] or a zip code associated with a Rural-Urban Commuting Area (RUCA) code of 4–10 [31]. All participants were enrolled within the first six months of initiating their current treatment and had a clinician defined life expectancy of at least six months. Full eligibility for the trial has been published elsewhere [29]. Potential participants were identified through the electronic medical record and permission to approach was given by the treating clinician. Patients were approached for consent and measurements at a regularly scheduled clinic visit between May 2022 and March 2025. All study activities were reviewed and approved by the WCG Institutional Review Board (www.wcgclinical.com) and the trial was registered with ClinicalTrials.gov as NCT05221606 on 27 January 2022. Trial reporting follows the CONSORT 2025 reporting guidelines (https://www.consort-spirit.org/).

### 2.1. Measurements

Demographic variables were collected using a self-report survey and included age, gender, marital status, race, ethnicity, education, employment, household number, and income. Clinical history related to cancer type, stage, and treatment were ascertained from the electronic medical record. Height and weight were collected from the medical record using clinic measurements on the day of consent. Body mass index (BMI) was calculated as weight in kilograms divided by height in meters squared. BMI was categorized as ‘normal weight’ (BMI ≤ 25 kg/m^2^), ‘overweight’ (BMI > 25 to 30 kg/m^2^), and ‘obese’ (BMI > 30 kg/m^2^).

Patient-reported health outcomes and health-related quality of life were assessed using the Patient-Reported Outcomes Measurement Information System Preference (PROMIS PROPr) survey [32] and the Medical Outcomes Study 36-item Short Form Survey (SF-36) [33]. The PROMIS domains include anxiety, depression, sleep, and cognition. The SF-36 subscales include general health perceptions, physical functioning, physical and emotional health problems, pain, emotional well-being, social functioning, and energy/fatigue. Subjective physical function was assessed using the physical functioning scale of the SF-36. The Short Physical Performance Battery (SPPB) was used to objectively assess physical function in-person by trained research staff. The SPPB includes a total score as well as sub scores for standing balance, gait speed, and repeated chair stands [34].

### 2.2. Statistical Analysis

Continuous measures were summarized with means and standard deviations and categorical measures were summarized with frequencies and percentages. Means for quantitative variables were compared among BMI groups with one-way ANOVA F-tests and proportions for categorical variables were compared with the chi-square or Fisher’s exact test. Covariates for adjustments were chosen according to between group differences with a *p*-value of 0.10 or less. Adjusted models utilized ANCOVA with covariates age, tumor type, and household number. All tests were two-sided and statistical significance was set at an alpha level of 0.05. Post hoc comparisons were adjusted with the Benjamini–Hochberg procedure. Statistical analyses were performed in SAS version 9.4.

## 3. Results

### 3.1. Sample Demographics

**Figure 1:**
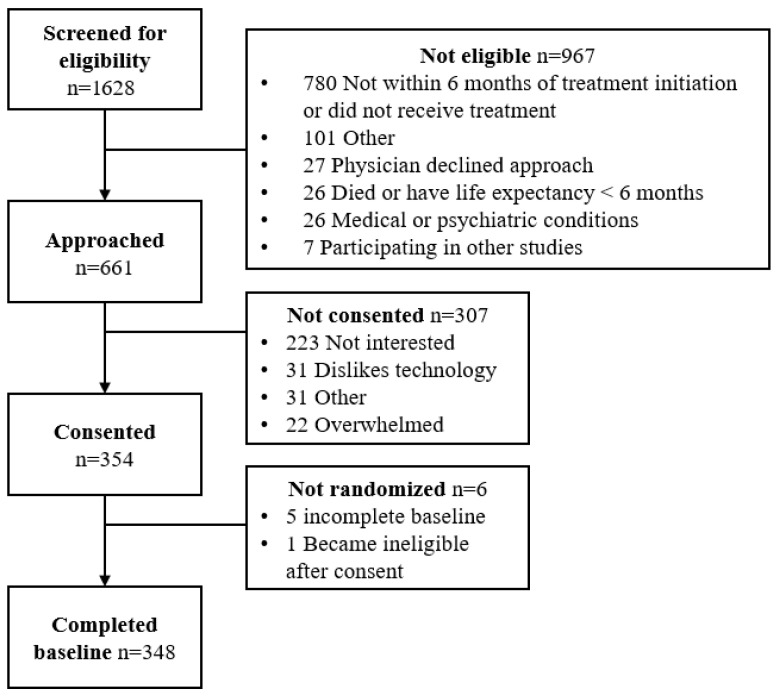
Participant flow chart.

A total of 348 advanced cancer patients were enrolled and randomized in the Nurse AMIE trial (Figure 1). Of these, 88 (25.3%) had a normal weight (BMI ≤ 25 kg/m^2^), 107 (30.7%) were overweight (BMI > 25 to 30 kg/m^2^), and 153 (44.0%) were classified as obese (BMI > 30 kg/m^2^) (Table 1). The mean BMI of the sample was 30.0 kg/m^2^ (SD = 7.2; Range: 14.3 to 56.9 kg/m^2^). The average age of participants at consent was 64.8 years (SD = 12.2) with no differences by BMI category at an alpha level of 0.05. Gender did not differ by BMI category with 46% (n = 160) of the sample overall being female. The majority of the sample identified as white (95%, n = 331).

Most participants were married or living with a partner (68%) and marital status did not differ significantly among BMI categories. The number of people living in the household varied among BMI groups (*p* < 0.0001). Prevalence of overweight and obesity did not differ by education or employment status with 49% (n = 171) having at least some college level education and 52% (n = 177) being retired. Annual household income did not differ between BMI categories with 43% (n = 149) of the sample having a household income of $50,000 or greater.

### 3.2. Clinical Characteristics

Cancer stage did not differ significantly among BMI categories with 32% (n = 110) of participants diagnosed in Stage 3, 52% (n = 182) diagnosed in Stage 4, and 16% (n = 56) diagnosed as advanced. Treatment modalities did not differ by BMI category. Most participants (88%, n = 307) received chemotherapy only. The most common cancer sub-types were colorectal (18%, n = 64), lung (17%, n = 58), hematologic (14%, n = 48), prostate (10%, n = 36), and breast (10%, n = 35) (Table 1).

### 3.3. Objective and Subjective Physical Function

Total scores for the SPPB were highest in the BMI less than or equal to 25 kg/m^2^ group and lowest in the BMI over 30 kg/m^2^ group (M ± SD: BMI ≤ 25 kg/m^2^: 9.05 ± 2.28; BMI > 25 to 30 kg/m^2^: 8.26 ± 3.05; BMI > 30 kg/m^2^: 8.12 ± 2.77; *p* = 0.04) (Table 2). A similar pattern was observed for the standing balance test, the repeated chair stands, and the SF-36 physical function subscore. Participants classified as normal weight had the highest mean scores followed by those in the overweight category and those classified as obese for both the standing balance test (M ± SD: BMI ≤ 25 kg/m^2^: 3.57 ± 0.72; BMI > 25 to 30 kg/m^2^: 3.23 ± 1.16; BMI > 30 kg/m^2^: 3.26 ± 1.05; *p* = 0.04) and the repeated chair stands (M ± SD: BMI ≤ 25 kg/m^2^: 2.30 ± 1.38; BMI > 25 to 30 kg/m^2^: 1.90 ± 1.43; BMI > 30 kg/m^2^: 1.79 ± 1.37; *p* = 0.02). Gait speed was not found to differ by BMI category. Similarly to the SPPB scores, participant self-reported physical function from the SF-36 was significantly lower across BMI categories (M ± SD: BMI ≤ 25 kg/m^2^: 57.93 ± 29.10; BMI > 25 to 30 kg/m^2^: 53.74 ± 27.96; BMI > 30 kg/m^2^: 47.59 ± 27.57; *p* = 0.02). These associations remained significant in models adjusted for age, tumor type, and household number (Table 2).

### 3.4. Health Outcomes and Quality of Life

No differences were detected in overall physical and emotional health; however, general health perceptions differed among BMI categories after adjustment for covariates. Participants classified as obese rated their general health lower than those with a normal or overweight BMI classification (M ± SD: BMI ≤ 25 kg/m^2^: 53.37 ± 23.88; BMI > 25 to 30 kg/m^2^: 53.37 ± 20.80; BMI > 30 kg/m^2^: 47.88 ± 21.18; *p* = 0.03). Additionally, participants with obesity reported lower levels of energy and greater fatigue compared to those with a normal or overweight BMI (M ± SD: BMI ≤ 25 kg/m^2^: 49.77 ± 26.11; BMI > 25 to 30 kg/m^2^: 45.10 ± 24.58; BMI > 30 kg/m^2^: 40.68 ± 22.60; unadjusted *p* = 0.02; adjusted *p* = 0.01). There were no differences in the pain, emotional well-being, or social functioning domains of the SF-36 or the PROMIS domains of anxiety, depression, sleep disturbance, and cognition.

## 4. Discussion

Our findings suggest that rural advanced cancer patients with higher BMI have worse physical function and higher fatigue. No associations were observed between BMI category and other patient-reported outcomes such as anxiety, sleep disturbance, and depression. Importantly, the high prevalence of people with a BMI greater than 25 kg/m^2^ in this sample (75%) underscores the need for targeted behavioral interventions that address physical function in rural cancer populations. Rural populations often face barriers to supportive care, including limited access to rehabilitation services, exercise programs, and nutrition counseling [26]. Tailored interventions may help address these barriers and mitigate obesity-related associations with poorer function and fatigue and improve overall quality of life.

The worse physical functioning among patients with a higher BMI observed in both the objective and subjective measures is clinically meaningful. Poor physical function not only impacts gross motor function, but also the level of functioning needed to live independently. Patients with lower reported physical function, particularly those who are self-reporting limited physical functioning, may have challenges with the Activities of Daily Living (ADL) like bathing, cooking, and cleaning. Up to one-half of cancer patients globally have been found to need assistance with ADLs, so decreased physical functioning associated with obesity may have an impact on caregiver burden and healthcare utilization [35]. For rural cancer patients who already face a barrier to accessing supportive care, this impact of obesity on physical function may be significant. Combined with the higher levels of fatigue that were observed in this cohort, these findings underscore the importance of addressing obesity to support and maintain mobility, balance, and coordination among rural cancer patients.

The lack of an association between obesity and psychosocial and patient-reported outcomes like anxiety, sleep disturbance, depression, and pain among patients in our sample needs further explanation. Previous research has largely compared rural versus urban populations concluding that rural cancer patients have higher rates of psychosocial distress [28,36]. When considering this intra-rural analysis, it is possible that the level of rurality or the level of access to supportive care services acts as a confounder to the association between obesity and psychosocial outcomes. This further supports the need for targeted interventions which consider not only the symptom profile of a patient, but also the complex social and contextual characteristics of rural life.

### 4.1. Implications and Future Directions

Findings from this work highlight the need for targeted interventions addressing obesity-related associations with poorer physical functioning and fatigue in rural cancer care. Poor physical function has significant implications for daily living, healthcare utilization, and quality of life among advanced cancer patients. Additional research should explore longitudinal patterns related to obesity and function among rural patients with advanced cancer to understand how obesity influences trajectories of physical function, fatigue, and health-related quality of life over time. Intervention development to address disparities in physical function in this population would benefit from qualitative work exploring patient perspectives on the barriers, facilitators, and contextual factors related to behavior change. Greater understanding of the characteristics of rural communities may better explain how social, environmental, and resource-related factors influence health behaviors and outcomes in rural populations and offer a more targeted approach for addressing this disparity.

### 4.2. Strengths and Limitations

This study offers a novel focus on the association between obesity and function among rural advanced cancer patients, a population which has had a dearth of research evidence related to health outcomes. The use of both objective and subjective measures of function increases the reliability and validity of outcome assessment. An additional strength is the use of clinical measures rather than participant self-report for BMI, increasing the validity of the findings. However, a limitation of utilizing BMI as a measure of body size is that it may not fully capture body composition or overall health. Another limitation to consider for this work is that cross-sectional data does not allow for determination of temporal or causal relationships, limiting the ability to infer directionality between observed associations. The generalizability of these findings is narrow since the sample is predominately white, relatively small, and from a single geographic region. There are other factors that may also contribute to poor function, such as comorbid conditions, which were not collected for this sample and should be explored in future work.

## 5. Conclusions

This study provides novel evidence that obesity in rural patients with advanced cancer may be associated with worse objective and subjective physical function and greater fatigue. Findings highlight the need for targeted interventions addressing physical function and fatigue in this high-risk population. These results underscore the importance of tailoring supportive care strategies to the specific symptom profiles influenced by obesity in rural cancer care.

## Figures and Tables

**Table 1:** Participant Characteristics, Overall and by BMI category.

Characteristic	Overall (N = 348)	BMI Category			*p*-Value
		≤25 kg/m^2^ (n = 88)	>25 to 30 kg/m^2^ (n =107)	>30 kg/m^2^ (n = 153)	
	No. (%)	No. (%)	No. (%)	No. (%)	
Age at consent in years, mean (SD)	64.8 (12.2)	65.4 (11.8)	66.6 (10.1)	63.2 (13.6)	0.08
Sex					
Female	160 (46)	48 (55)	39 (36)	73 (48)	0.12
Male	180 (52)	38 (43)	65 (62)	77 (50)	
Unknown	8 (2)	2 (2)	3 (3)	3 (2)	
Race					
Non-white	10 (3)	3 (3)	3 (3)	4 (3)	0.92
White	331 (95)	83 (94)	102 (95)	146 (95)	
Unknown	7 (2)	2 (2)	2 (2)	3 (2)	
Current marital status					
Single or never married	28 (8)	3 (3)	8 (7)	17 (11)	0.53
Currently married or living with a partner	237 (68)	58 (66)	74 (69)	105 (69)	
Divorced or separated	47 (14)	15 (17)	15 (14)	17 (11)	
Widowed or widower	29 (8)	10 (11)	8 (7)	11 (7)	
Unknown	7 (2)	2 (2)	2 (2)	3 (2)	
Number living in household					
One	54 (16)	15 (17)	20 (19)	19 (12)	<0.0001 *
Two	191 (55)	42 (48)	73 (68)	76 (50)	
More than 2	95 (27)	29 (33)	11 (10)	55 (36)	
Unknown	8 (2)	2 (2)	3 (3)	3 (2)	
Number contributing to household income					
Zero	7 (2)	2 (2)	2 (2)	3 (2)	0.79
One	92 (26)	26 (30)	25 (23)	41 (27)	
Two	220 (63)	51 (58)	74 (69)	95 (62)	
More than 2	21 (6)	7 (8)	3 (3)	11 (7)	
Unknown	8 (2)	2 (2)	3 (3)	3 (2)	
Educational attainment					
9th–11th grade	21 (6)	4 (5)	4 (4)	13 (8)	0.76
HS graduate/GED	149 (43)	40 (45)	46 (43)	63 (41)	
Some college	99 (28)	23 (26)	29 (27)	47 (31)	
College graduate or post graduate degree	72 (21)	19 (22)	26 (24)	27 (18)	
Unknown	7 (2)	2 (2)	2 (2)	3 (2)	
Employment					
Working now	66 (19)	15 (17)	20 (19)	31 (20)	0.49
Only temporarily laid off, on sick leave, or on maternity leave	16 (5)	2 (2)	6 (6)	8 (5)	
Looking for work or unemployed	2 (1)	1 (1)	1 (1)	0 (0)	
Retired	177 (52)	50 (57)	61 (57)	66 (43)	
Disabled, permanently or temporarily	58 (17)	13 (15)	13 (12)	32 (21)	
Keeping house	9 (3)	2 (2)	2 (2)	5 (3)	
Other or Unknown	20 (6)	5 (6)	3 (4)	11 (7)	
Annual household income (pre-tax)					
Less than $10,000	17 (5)	5 (6)	6 (6)	6 (4)	0.79
$10,000 to $24,999	53 (15)	14 (16)	19 (19)	20 (13)	
$25,000 to $49,999	90 (26)	24 (27)	31 (29)	35 (23)	
$50,000 to $74,999	75 (22)	20 (23)	18 (17)	37 (24)	
$75,000 to $99,999	38 (11)	6 (7)	12 (11)	20 (13)	
$100,000 and greater	36 (10)	7 (8)	10 (9)	19 (12)	
Unknown	39 (12)	12 (14)	11 (10)	16 (11)	
Stage of cancer					
Stage 3	110 (32)	24 (27)	41 (38)	45 (29)	0.33
Stage 4	182 (52)	52 (59)	50 (47)	80 (52)	
Advanced	56 (16)	12 (14)	16 (15)	28 (18)	
Cancer treatment					
Chemotherapy	307 (88)	76 (86)	92 (86)	139 (91)	0.36
Radiation	10 (3)	3 (3)	2 (2)	5 (3)	
Chemotherapy and Radiation	23 (7)	8 (9)	10 (9)	5 (3)	
Other	8 (2)	1 (1)	3 (3)	4 (3)	
Tumor type					0.002
Breast	35 (10)	5 (6)	11 (10)	19 (12)	
Colorectal	64 (18)	18 (20)	20 (19)	26 (17)	
Esophageal	12 (3)	5 (6)	5 (5)	2 (1)	
Head and neck	11 (3)	6 (7)	2 (2)	3 (2)	
Hematologic	48 (14)	14 (16)	13 (12)	21 (14)	
Lung	58 (17)	16 (18)	25 (23)	17 (11)	
Melanoma	10 (3)	0 (0)	3 (3)	7 (5)	
Ovarian	13 (4)	2 (2)	5 (5)	6 (4)	
Pancreas	14 (4)	9 (10)	1 (1)	4 (3)	
Prostate	36 (10)	2 (2)	12 (11)	22 (14)	
Renal	11 (3)	1 (1)	3 (3)	7 (5)	
Other	36 (10)	10 (11)	7 (7)	19 (12)	
Body mass index ^a^ (kg/m^2^), mean (SD)	30.0 (7.2)	22.1 (2.2)	27.4 (1.4)	36.5 (5.5)	
RUCC ≥ 7	9 (3)	2 (2)	3 (3)	4 (3)	1.00
RUCA ≥ 7	85 (24)	17 (19)	30 (28)	38 (25)	0.37

Continuous variables compared between groups with one-way ANOVA F-test. Categorical variables compared with the chi-square or Fisher’s exact test. ^a^ Calculated as weight in kilograms divided by height in meters squared. * *p*-value comparing 25 or under to >25 to 30 = 0.002, *p*-value comparing 25 or under to >30 = 0.77, *p*-value comparing >25 to 30 to >30 = 0.0003 (post hoc comparisons adjusted with the Benjamini–Hochberg procedure).

**Table 2:** Function, Symptoms, and Quality of Life, Overall and by BMI category.

Variable	Overall (N = 348)		BMI Category						*p* Value ^a^	*p* Value ^b^
			≤25 kg/m^2^ (n = 88)		>25 to 30 kg/m^2^ (n =107)		>30 kg/m^2^ (n = 153)			
	No.	Mean (SD)	No.	Mean (SD)	No.	Mean (SD)	No.	Mean (SD)		
PROMIS ^c^										
Anxiety	335	51.24 (9.47)	84	52.03 (10.03)	102	49.95 (8.66)	149	51.67 (9.64)	0.24	0.78
Depression	333	49.13 (8.86)	85	48.78 (9.16)	103	48.12 (8.55)	145	50.04 (8.87)	0.22	0.21
Sleep	331	51.63 (7.67)	83	50.83 (8.11)	99	50.65 (6.53)	149	52.71 (8.01)	0.06	0.06
Cognition	330	50.26 (8.38)	85	50.69 (8.61)	99	50.80 (8.16)	146	49.66 (8.41)	0.50	0.64
SPPB ^d^										
SPPB total score	347	8.40 (2.77)	88	9.05 (2.28)	106	8.26 (3.05)	153	8.12 (2.77)	0.04	0.01
Standing balance test	347	3.33 (1.02)	88	3.57 (0.72)	106	3.23 (1.16)	153	3.26 (1.05)	0.04	0.04
Gait speed score	347	3.12 (1.18)	88	3.18 (1.05)	106	3.14 (1.28)	153	3.07 (1.18)	0.74	0.57
Repeated chair stands	347	1.95 (1.40)	88	2.30 (1.38)	106	1.90 (1.43)	153	1.79 (1.37)	0.02	0.003
SF-36 ^e^										
Physical functioning	340	52.09 (28.33)	86	57.93 (29.10)	104	53.74 (27.96)	150	47.59 (27.57)	0.02	0.004
Physical health problems	340	31.13 (40.34)	86	35.85 (43.63)	104	30.29 (39.67)	150	29.00 (38.87)	0.44	0.42
Pain	340	63.13 (26.45)	86	66.19 (27.62)	104	64.88 (25.57)	150	60.17 (26.24)	0.17	0.19
General health perceptions	340	50.94 (21.89)	86	53.37 (23.88)	104	53.37 (20.80)	150	47.88 (21.18)	0.07	0.03
Emotional well-being	338	73.59 (19.30)	86	75.07 (18.75)	103	74.72 (18.73)	149	71.96 (20.00)	0.38	0.09
Emotional health problems	339	66.18 (41.60)	85	69.02 (40.76)	104	70.03 (40.78)	150	61.89 (42.50)	0.24	0.25
Social functioning	340	63.86 (27.97)	86	63.95 (29.54)	104	65.87 (28.84)	150	62.42 (26.49)	0.63	0.16
Energy/fatigue	338	44.34 (24.34)	86	49.77 (26.11)	103	45.10 (24.58)	149	40.68 (22.60)	0.02	0.01

Abbreviations: PROMIS, Patient-Reported Outcomes Measurement Information System; SPPB, Short Physical Performance Battery; SF-36, Medical Outcomes Study 36-Item Short Form Survey. ^a^ One-way ANOVA. ^b^ ANCOVA with covariates age, tumor type, and household number. ^c^ Scale of 0 to 100 with higher values indicating greater prevalence of the symptom. ^d^ Scale of 0 to 12 for the total score and 0 to 4 for the subscales with higher values indicating greater function. ^e^ Scale of 0 to 100 with higher values indicating greater function except for the pain scale which is reversed.

## Data Availability

The data that support the findings of this study are available from the corresponding author upon reasonable request.

## Abbreviations

The following abbreviations are used in this manuscript:

BMI	Body Mass Index
SPPB	Short Physical Performance Battery
PROMIS	Patient-Reported Outcomes Measurement Information System Preference
SF-36	Medical Outcomes Study 36-item Short Form Survey
RUCC	Rural Urban Continuum Code
RUCA	Rural-Urban Commuting Area
